# Reproducibility of flutter-range vibrotactile detection and discrimination thresholds

**DOI:** 10.1038/s41598-020-63208-z

**Published:** 2020-04-16

**Authors:** Mark Mikkelsen, Jason He, Mark Tommerdahl, Richard A. E. Edden, Stewart H. Mostofsky, Nicolaas A. J. Puts

**Affiliations:** 1 Russell H. Morgan Department of Radiology and Radiological Science, The Johns Hopkins University School of Medicine, Baltimore, MD USA; 2 F. M. Kirby Research Center for Functional Brain Imaging, Kennedy Krieger Institute, Baltimore, MD USA; 3 Department of Biomedical Engineering, University of North Carolina at Chapel Hill, Chapel Hill, NC USA; 4 Center for Neurodevelopmental and Imaging Research, Kennedy Krieger Institute, Baltimore, MD USA; 5 Department of Neurology, The Johns Hopkins University School of Medicine, Baltimore, MD USA; 6 Department of Psychiatry and Behavioral Sciences, The Johns Hopkins University School of Medicine, Baltimore, MD USA; 7 Department of Forensic and Neurodevelopmental Sciences, Sackler Institute for Translational Neurodevelopment, Institute of Psychiatry, Psychology, and Neuroscience, King’s College London, London, UK

**Keywords:** Sensory processing, Cortex

## Abstract

Somatosensory processing can be probed empirically through vibrotactile psychophysical experiments. Psychophysical approaches are valuable for investigating both normal and abnormal tactile function in healthy and clinical populations. To date, the test-retest reliability of vibrotactile detection and discrimination thresholds has yet to be established. This study sought to assess the reproducibility of vibrotactile detection and discrimination thresholds in human adults using an established vibrotactile psychophysical battery. Fifteen healthy adults underwent three repeat sessions of an eleven-task battery that measured a range of vibrotactile measures, including reaction time, detection threshold, amplitude and frequency discrimination, and temporal order judgement. Coefficients of variation and intraclass correlation coefficients (ICCs) were calculated for the measures in each task. Linear mixed-effects models were used to test for length and training effects and differences between tasks within the same domain. Reaction times were shown to be the most reproducible (ICC: ~0.9) followed by detection thresholds (ICC: ~0.7). Frequency discrimination thresholds were the least reproducible (ICC: ~0.3). As reported in prior studies, significant differences in measures between related tasks were also found, demonstrating the reproducibility of task-related effects. These findings show that vibrotactile detection and discrimination thresholds are reliable, further supporting the use of psychophysical experiments to probe tactile function.

## Introduction

Psychophysical experiments can be used to probe somatosensory processing empirically. Quantitative measures of vibrotactile sensitivity, in particular, have been used to elucidate on the cortical mechanisms underlying such processing^1,2^. Such behavioural approaches have proven to be valuable and may reflect cortical mechanisms underlying somatosensory function. For example, GABAergic inhibition drives neuronal responses to sensory stimulation^3,4^ linked to behavioural outcomes. Measuring such behavioural outcomes may, therefore, provide information about cortical inhibition. The targeting of specific neuronal mechanisms of tactile function through psychophysics allows for the investigation of individual differences in healthy brain function or of impairments in disorders hypothesized to be driven by inhibitory dysfunction. Early work established the neurophysiological basis of tactile function in both nonhuman and human primates^5–10^. Psychophysical testing of sensory processing in general also has a long history^11,12^ upon which present-day research has been built.

A behavioural battery of psychophysical paradigms for assessing vibrotactile function^13^ was previously developed and shown to be successfully implementable in both adults and typically developing children. The vibrotactile stimuli used in the paradigms fall within the flutter range of touch (<50 Hz), which are processed by rapidly adapting (RA) I mechanoreceptors in the glabrous skin^7,9^. The neurophysiology of amplitude and frequency discrimination of low-frequency stimuli is well-characterized^1,8,14,15^, which has engendered the investigation of the neural coding linking sensation and complex cognition^16^. Thus, psychophysical testing using flutter-range stimuli permits interrogation of the somatosensory and higher-level systems in healthy and pathological neurobiology. This battery and its variants have been used to investigate tactile (dys)function in autism spectrum disorder^17–22^, Tourette syndrome^23^, and attention-deficit hyperactivity disorder^24^; in ageing^25,26^; in multimodal studies involving magnetic resonance spectroscopic measures of GABA^27–29^; in concussion^30,31^; and in conjunction with neurostimulation^32^. Dynamic detection thresholds, for example, are thought to reflect local feed-forward inhibitory mechanisms^33–35^. On the other hand, using an amplitude discrimination paradigm to present two stimuli simultaneously can probe lateral inhibition, where an adapting stimulus presented beforehand can sharpen the resulting response function^15,36^.

Despite the extensive use of vibrotactile stimulation paradigms in the literature, the reproducibility of detection and discrimination thresholds is as yet unknown. Determining their reproducibility is essential for the validation of past and future findings from basic science and clinical studies. It is also not well known whether these measures represent states or traits. Furthermore, as psychophysical experiments typically involve relatively long test sessions, it is worthwhile to investigate whether paradigms can be shortened without loss in reliability so that they may be applicable in clinical settings. Therefore, this study aimed to assess the test-retest reliability of vibrotactile detection and discrimination thresholds in healthy adults using an established vibrotactile processing battery comprised of paradigms (in both short and long forms) designed to target different aspects of tactile processing.

## Methods

### Participants

Fifteen adult participants (8 females/7 males) were recruited (mean age: 28.3 ± 3.9 years; age range: 21–37 years). Participants were required to meet the following eligibility criteria to take part in experiments: aged 18–40 years; no history of a clinically diagnosed neurological or psychiatric disorder; no history of concussion; right-handed; non-smoker; no recreational drug use (alcohol was permissible); not colour-blind; no stimulant medication use (birth control was permissible); educated to a high-school diploma level (or the equivalent) or higher. All criteria (except for handedness) were confirmed verbally by participants. All volunteers were right-handed, which was confirmed using the Edinburgh Handedness Inventory^37^. This study was approved by the Johns Hopkins Medicine Institutional Review Board and was performed in accordance with all relevant institutional guidelines and federal regulations. All participants provided written informed consent prior to their participation in the study.

Participants were tested three times over an approximately three-week period to test the reproducibility of their vibrotactile detection and discrimination thresholds. To the extent that was practicable given time constraints and participants’ availability, repeat sessions occurred approximately one week after another (mean session-to-session interval: 7.2 ± 2.0 days; interval range: 4–15 days).

### Stimulus delivery

A CM-4 four-digit vibrotactile stimulator^38^ (Cortical Metrics, Carrboro, NC) was used for stimulus delivery. All stimuli were delivered to the glabrous skin of the left digits 2 and 3 (LD2 and LD3) using cylindrical probes (5-mm diameter) and presented within the flutter range (25–50 Hz) using sinusoidal pulses. The stimulator operates with 16-bit resolution and has a displacement accuracy of less than 1 μm. Its temporal accuracy is less than 1 ms^39^. Visual feedback, task responses and data collection were performed using a Dell Inspiron Mini laptop running Cortical Metrics software^38^ in Windows 7. Participants responded by clicking on the left or right buttons of a mouse using their right hand. The left mouse button corresponded to LD3 and the right mouse button correspond to LD2. In all tasks, stimuli were delivered pseudorandomly to LD2 and LD3. Great efforts were made in the design and fabrication of the vibrotactile stimulator to minimize any detectable noise. The internal mechanism of the CM-4 head unit is driven by a voice coil actuator and does not produce any audible cues during tactile stimulation^38^. This was confirmed from experimental data collected from three additional participants (see Data Availability statement for access to these results).

### Vibrotactile paradigms

**Figure 1:**
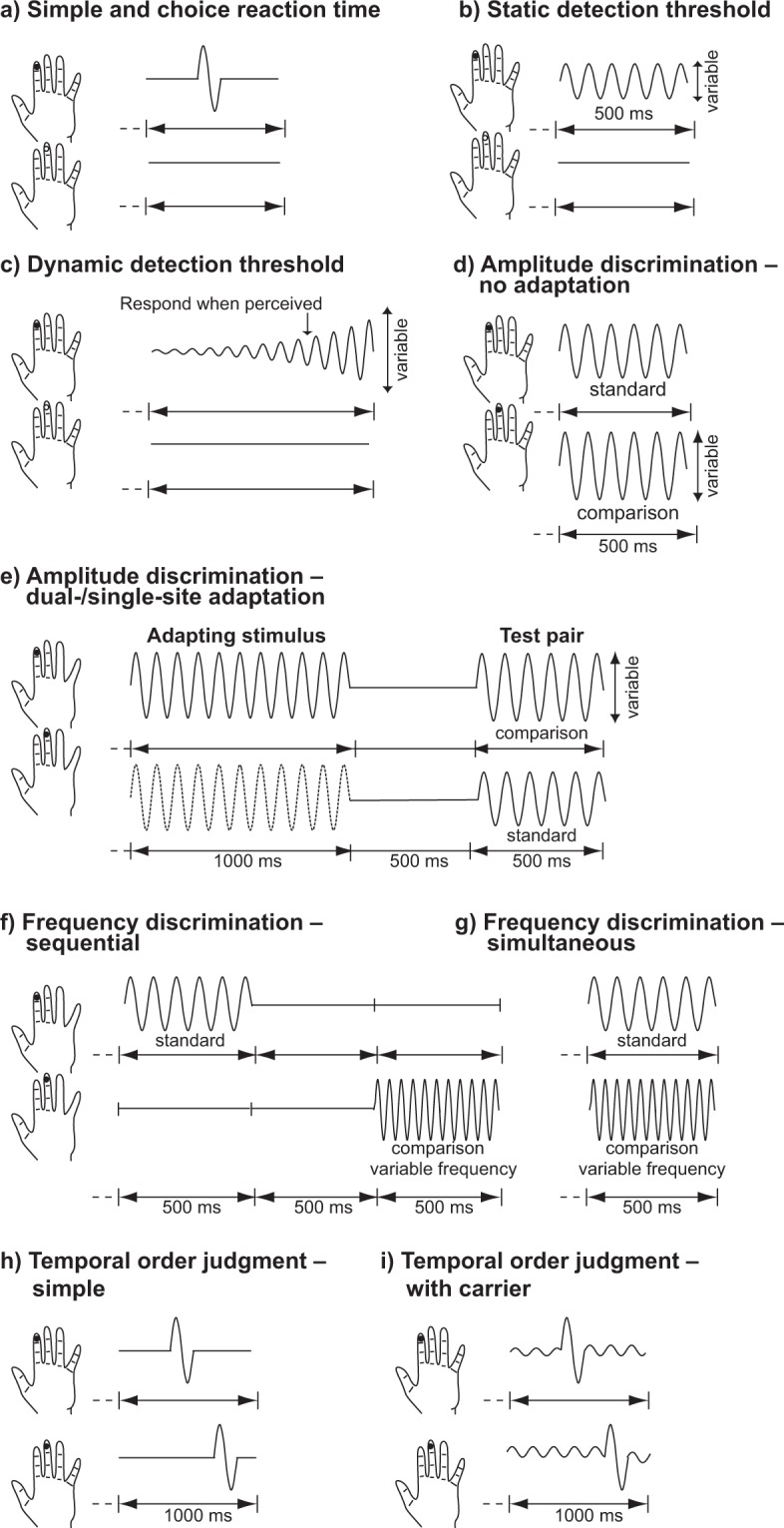
Schematic of the vibrotactile paradigms. (**a**) Simple and choice reaction time; (**b**) static detection threshold; (**c**) dynamic detection threshold; (**d**) amplitude discrimination with no adaptation; (**e**) amplitude discrimination with dual-/single-site adaptation; (**f**) sequential frequency discrimination; (**g**) simultaneous discrimination; (**h**) temporal order judgement; and (**i**) temporal order judge with carrier stimulus.

A previously published vibrotactile battery^13^ consisting of 11 separate paradigms was used in this study. The paradigms are illustrated in Fig. 1. Each task was preceded by three practice trials to familiarize participants with the goal of the specific paradigm. Participants were required to successfully complete all three trials to proceed to the testing component. Feedback was provided during training but not during the testing component. Participants underwent two versions of the battery: a short and long version. Each task in the short version had the same number of trials compared to the work published previously^13^ and was delivered twice over the three sessions (denoted Short(1) and Short(2)); tasks in the long version had twice the number of trials compared to the short, original, version, and was delivered only once over the three sessions (denoted Long). The short version took approximately 40 min to complete, while the long version took approximately 1 hr. Session order was randomized across participants. This experimental design allowed us to study the effect of version (Short vs. Long) and effect of training (week effect). Finally, the long version could be truncated to reflect a “Short(3)” measurement.

#### Simple (sRT) and choice (cRT) reaction time

A suprathreshold stimulus (frequency = 25 Hz; amplitude = 300 μm; duration = 40 ms) was delivered to LD2 or LD3, and participants were ﻿asked to respond as soon as they felt the stimulus (Fig. 1a). In the sRT task, participants simply needed to click any mouse button, whereas in the cRT task participants additionally had to indicate, using the left and right mouse buttons, on which finger they felt the stimulus (intertrial interval (ITI) = 3 s; Short(1) and Short(2): 20 trials; Long: 40 trials). For each individual, reaction times (for correct trials only in the cRT task) were calculated as the median reaction time. Intrasubject variability (ISV) was also calculated as the standard deviation of the values from all trials (outliers were removed using the method described in “Statistical analysis” below).

#### Static (sDT) and dynamic (dDT) detection threshold

In the sDT task, a suprathreshold stimulus (frequency = 25 Hz; starting amplitude = 20 μm; duration = 500 ms) was delivered to one of the two ﻿digits and participants were asked to respond on which finger they felt the stimulus (Fig. 1b,c). A one-up–one-down tracking paradigm (stimulus amplitude was decreased for a correct answer and increased for an incorrect answer) was used for the first 10 trials and a two-up–one-down (two correct answers were necessary for a reduction in test amplitude) was used for the remainder of the task (ITI = 5 s; Short(1) and Short(2): 24 trials; Long: 48 trials). sDT was calculated as the mean of the amplitudes of the last five values. In the dDT task, after a variable delay (0–2500 ms), a 25-Hz stimulus increased from zero amplitude (rate of amplitude increase = 2 μm/s). Participants were asked to respond as soon as they felt the stimulus and on which finger they felt it (ITI = 10 s; Short(1) and Short(2): 7 trials; Long: 14 trials). dDT was calculated as the mean stimulus amplitude at the time of pressing the button, across all correct trials. These thresholds were not corrected for reaction time. As was the case for all subsequent tasks, convergence scores were calculated as the accuracy of performance in the final five trials (i.e., the trials used to estimate thresholds).

#### Amplitude discrimination with no adaptation (nAD), dual-site adaptation (dAD) and single-site adaptation (sAD)

In the nAD task, participants were asked to choose which of two simultaneously delivered stimuli had the higher amplitude (frequency = 25 Hz; duration = 500 ms; standard stimulus amplitude = 100 μm; initial comparison stimulus amplitude = 200 μm) (Fig. 1d,e). In the dAD condition, each trial was preceded by dual-site-delivered adapting stimuli (frequency = 25 Hz; duration = 1 s, amplitude = 100 μm) and in the sAD task each trial was preceded by a single-site-delivered adapting stimulus (duration = 1 s, amplitude = 100 μm). The single-site adapting stimulus was presented to the same digit that received the test stimulus of higher amplitude. A one-up–one-down tracking paradigm (comparison stimulus amplitude was decreased for a correct answer and increased for a wrong answer) was used for the first 10 trials and a two-up–one-down (two correct answers were necessary for a reduction in comparison stimulus amplitude) was used for the remainder of the task (ITI = 5 s; Short(1) and Short(2): 20 trials; Long: 40 trials). Amplitude discrimination thresholds were calculated as the mean of the amplitudes of the last five values. In the Long nAD task, the initial comparison stimulus was inadvertently set to 300 μm. While inconsistent with the short versions of the task, this allowed for an investigation of nAD thresholds between short and long versions where the long version had a larger range of stimulus parameters (initial difference between stimuli = 200 μm).

#### Sequential (sqFD) and simultaneous (smFD) frequency discrimination

In the sqFD task, stimuli (duration = 500 ms; amplitude = 200 μm) were delivered to LD2 and LD3 sequentially (interstimulus interval (ISI) = 500 ms) (Fig. 1f,g). In the smFD task, the two stimuli were delivered simultaneously to both digits. One finger always received the standard stimulus (frequency = 30 Hz) while the other received the comparison stimulus (initial frequency = 40 Hz). The two stimuli were delivered to either location pseudorandomly. In both conditions, the participants were asked which finger received the higher frequency stimulus. An one-up–one-down tracking paradigm (the comparison stimulus frequency was decreased for a correct answer and increased for a wrong answer) was used for the first 10 trials and the two-up–one-down (two correct answers were necessary for a reduction in comparison stimulus frequency) was used for the remainder of the task (ITI = 5 s; Short(1) and Short(2): 20 trials; Long: 40 trials). Frequency discrimination thresholds were obtained as the mean of the frequencies of the last five trials.

#### Temporal order judgment without (TOJs) and with carrier stimulus (TOJc)

In this task, two single-cycle vibrotactile pulses (duration = 40 ms; frequency = 25 Hz; amplitude = 200 μm) were delivered to LD2 and LD3 separated temporally by a starting ISI of 150 ms (the first pulse was assigned to either digit pseudorandomly) within a 1-s interval (Short(1) and Short(2): 20 trials; Long: 40 trials) (Fig. 1h,i). Participants were asked to respond which digit received the first pulse. TOJ thresholds were calculated as the mean of the ISI of the last five trials. In the TOJs condition, there was no concurrent stimulation, while in the TOJc condition a 25-Hz, 20-μm concurrent carrier stimulus was delivered throughout each 1-s trial interval.

### Statistical analysis

All statistical analyses were performed in R^40^ (version 3.5.3). For each task, outliers were first detected using the median absolute deviation method^41^ with a threshold value of 2.5^42^ by collapsing measurements from all three sessions together to better estimate the dispersion of the measurements. All results are presented with outliers removed. A repository for the raw data can be accessed on the Open Science Framework website (see Data Availability statement).

Between-subject (CV_bs_) (both within session and over all sessions) and within-subject (CV_ws_) coefficients of variation were calculated for each task:

1 $${{\rm{CV}}}_{{\rm{bs}}}=100\frac{{\sigma }_{g}}{{\mu }_{g}}$$

2 $${{\rm{CV}}}_{{\rm{ws}}}=\frac{1}{n}\mathop{\sum }\limits_{s=1}^{n}100\frac{{\sigma }_{s}}{{\mu }_{s}}$$

where σ_*g*_ and μ_*g*_ are the group standard deviations and means across subjects’ measurements, either within each session or over all three sessions (*g*); σ_*s*_ and μ_*s*_ are the standard deviation and mean of measurements across the three sessions for subject *s*. CVs are useful as they provide a standardized estimate of intra- and interindividual variability that can be compared across different measures. To assess test-retest reliability, intraclass correlation coefficients (ICCs) were calculated. The ICC is a relative measure of reliability and is formulated as:

3 $${\rm{ICC}}=\frac{{\sigma }_{g}^{2}}{{\sigma }_{g}^{2}+{\sigma }_{e}^{2}}$$

where ${\sigma }_{g}^{2}$ is the between-subjects variance and ${\sigma }_{e}^{2}$ is the error variance, which includes biological or state variability, errors from subjects, and errors from the instrumentation or tester. As the ICC is driven by between-subjects variance, a large value indicates that the variability in a measure is predominately driven by individual differences (i.e., the true variance) rather than trial-to-trial variability that is due to error (i.e., the difference between the true variance and the observed variance). Several versions of the ICC exist; in the present study, the ICC was calculated using a two-way mixed-effects model with average measures of absolute agreement^43,44^ using the *psych* R package^45^.

Repeated-measures linear mixed-effects modelling was performed using the *lme4* R package^46^ (using maximum likelihood for parameter estimation) to test (i) whether there were significant differences between the short and long versions of each task (a version effect), (ii) whether there were training effects within task (a time effect), and (iii) whether there were significant differences between tasks within a domain (a task effect). This is formulated as:

4 $$\begin{array}{c}{y}_{ij}={\beta }_{0}+{s}_{j}+{\beta }_{1}{x}_{ij}+{\varepsilon }_{ij}\\ {s}_{j}\, \sim \,N(0,{\sigma }_{s}^{2})\\ {\varepsilon }_{ij}\, \sim \,N(0,{\sigma }_{\varepsilon }^{2})\end{array}$$

where *y*_*ij*_ is the threshold measurement of a given task (or tasks within the same domain) for subject *j* from session *i*; β_0_ is the model intercept (the grand mean); *s*_*j*_ is the by-subject random effect (which accounts for variation across subjects) with mean 0 and variance ${\sigma }_{s}^{2}$; *x*_*ij*_ is the predictor variable (i.e., the fixed effect of version, time, or task) with a grand mean slope of β_1_; and ε_*ij*_ is the residual error with mean 0 and variance ${\sigma }_{\varepsilon }^{2}$. For these analyses, Short(1) and Short(2) threshold measurements were collapsed in the mixed-effects models. ﻿Goodness-of-fit was calculated as a log-likelihood statistic. ﻿To test for significant effects, likelihood ratio tests were performed by comparing the log-likelihood statistic of one model to that of a reduced model (i.e., a model excluding the effect of interest). The alpha level was set at 0.05. Effect sizes were calculated as the proportional reduction in residual variance between the reduced model and the model of interest^47^. Where applicable, two-tailed post hoc comparisons were performed using Tukey’s HSD test. Multiple comparisons were corrected for Type I error rate inflation using the Holm-Bonferroni method^48^ using the *multcomp* R package^49^ (adjusted *p*-values are denoted: *p*_Holm_).

To test whether shortening the long version of the paradigms improved the test-retest reliability of detection and discrimination thresholds, a secondary analysis was performed where the number of recorded trials for the long version were truncated to the same number of trials of the short version and thresholds were subsequently calculated on the basis of the last five trials. CVs and ICCs were then recalculated and compared to those from the primary analysis.

To test whether there were any differences of convergence in the paradigms where staircase tracking was implemented, a convergence score was calculated as the accuracy of performance in the final five trials (i.e., the trials used to estimate thresholds). A convergence score of 5 would suggest that the participant did not make any inaccurate responses in the final five trials, whereas a score of 0 would mean that the participant did not make an accurate response in the final five trials. Note that these scores were not used to indicate whether convergence was good or bad. Rather, they simply allowed us to determine whether convergence was comparable between the tasks, versions, and sessions. While it is presumable that a convergence score of 0 or 5 rather than 3 would suggest worse convergence, a consensus on what is considered an acceptable level of convergence has not yet been established. Since the scope of this study was to assess the reproducibility of flutter-range tactile detection and discrimination thresholds, rather than reliability of convergence, only group comparisons of the convergence between tasks, versions and sessions is provided. To this end, convergence between the protocols was compared using repeated-measures linear mixed-effects modelling using the methods described above to determine whether there was (i) a task effect, (ii) a version effect, (iii) a time effect, (iii) a task × version effect, and (iv) a task × time effect.

## Results

**Figure 2:**
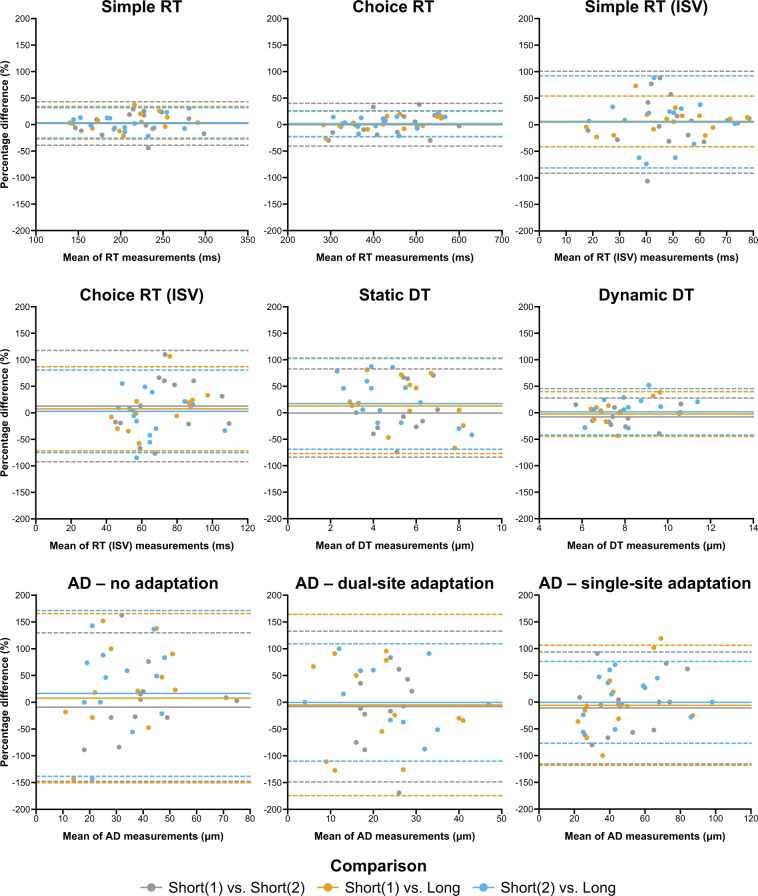
Bland-Altman plots visualizing the pairwise agreement between each participant’s measurements between versions (i.e., Short(1) vs. Short(2) (grey), Short(1) vs. Long (orange), and Short(2) vs. Long (blue)) for simple and choice reaction time (RT), simple and choice RT intrasubject variability (ISV), static and dynamic detection threshold (DT), and amplitude discrimination (AD) with no, dual-site, and single-site adaptation. The comparisons are overlaid within each subplot to better illustrate the agreement across all three comparisons.

**Figure 3:**
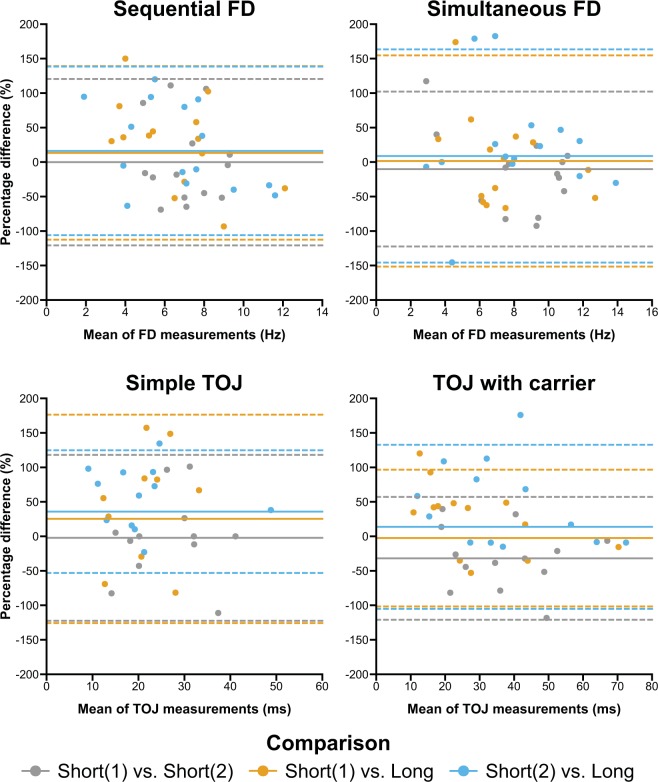
Bland-Altman plots for sequential and simultaneous frequency discrimination (FD) and temporal order judgment (TOJ) without and with carrier stimulus. Plot details as in Fig. 2.

All participants were able to complete the 11 paradigms successfully. Table 1 summarizes the behavioural results for each paradigm, including the mean (±1 standard deviation) vibrotactile detection and discrimination thresholds, CV_bs_, CV_ws_ and ICCs. Bland-Altman plots displaying the agreement between participants’ repeated measurements are shown in Figs. 2 and 3. These are presented as the mean of pairs of measurements (e.g., the mean of Short(1) and Short(2) thresholds) versus their percentage difference (e.g., [Short(1) – Short(2) thresholds] / mean of Short(1) and Short(2) thresholds × 100). Table 2 shows the results following truncation of the long version of each paradigm.

**Table 1:** Descriptive statistics of results from the vibrotactile tasks.

Task	Session	Mean ± SD	CV_bs_	CV_ws_	ICC
sRT	Short(1)	217.75 ± 51.34 ms	23.58%	10.85%	0.90
	Short(2)	212.46 ± 49.09 ms	23.11%		
	Long	216.10 ± 56.05 ms	25.94%		
	**All sessions**	215.45 ± 51.13 ms	23.73%		
sRT (ISV)	Short(1)	50.42 ± 19.07 ms	37.82%	23.41%	0.78
	Short(2)	47.67 ± 18.52 ms	38.85%		
	Long	46.06 ± 17.57 ms	38.15%		
	**All sessions**	48.05 ± 18.07 ms	37.60%		
cRT	Short(1)	434.83 ± 112.31 ms	25.83%	9.50%	0.90
	Short(2)	430.87 ± 93.13 ms	21.62%		
	Long	417.10 ± 78.64 ms	18.85%		
	**All sessions**	427.60 ± 93.81 ms	21.94%		
cRT (ISV)	Short(1)	78.55 ± 30.51 ms	38.84%	26.53%	0.63
	Short(2)	67.71 ± 23.70 ms	35.00%		
	Long	73.61 ± 31.01 ms	42.13%		
	**All sessions**	73.17 ± 28.24 ms	38.59%		
sDT	Short(1)	5.51 ± 2.10 μm	38.19%	30.85%	0.65
	Short(2)	5.17 ± 1.45 μm	28.01%		
	Long	4.61 ± 2.70 μm	58.45%		
	**All sessions**	5.10 ± 2.14 μm	42.01%		
dDT	Short(1)	7.94 ± 1.90 μm	23.95%	13.48%	0.78
	Short(2)	9.03 ± 2.16 μm	23.94%		
	Long	7.93 ± 1.38 μm	17.45%		
	**All sessions**	8.32 ± 1.87 μm	22.47%		
nAD	Short(1)	38.13 ± 21.74 μm	57.02%	40.45%	0.44, 0.68*
	Short(2)	37.87 ± 18.01 μm	47.55%		
	Long	25.23 ± 13.63 μm	54.01%		
	**All sessions**	34.14 ± 18.81 μm	55.11%		
dAD	Short(1)	19.86 ± 11.83 μm	59.58%	45.71%	0.53
	Short(2)	23.29 ± 11.65 μm	50.04%		
	Long	22.71 ± 15.00 μm	66.04%		
	**All sessions**	21.95 ± 12.69 μm	57.81%		
sAD	Short(1)	50.27 ± 29.29 μm	58.26%	31.44%	0.72
	Short(2)	52.27 ± 22.46 μm	42.97%		
	Long	46.67 ± 23.12 μm	49.55%		
	**All sessions**	49.73 ± 24.68 μm	49.62%		
sqFD	Short(1)	7.03 ± 2.50 Hz	35.63%	41.72%	0.25
	Short(2)	7.15 ± 2.59 Hz	36.19%		
	Long	6.40 ± 4.04 Hz	63.12%		
	**All sessions**	6.85 ± 3.08 Hz	44.96%		
smFD	Short(1)	7.37 ± 2.59 Hz	35.08%	36.96%	0.43
	Short(2)	8.97 ± 3.87 Hz	43.13%		
	Long	7.12 ± 4.15 Hz	58.22%		
	**All sessions**	7.82 ± 3.61 Hz	46.18%		
TOJs	Short(1)	27.79 ± 12.79 ms	46.02%	45.94%	0.07
	Short(2)	26.52 ± 13.06 ms	49.25%		
	Long	18.12 ± 11.08 ms	61.17%		
	**All sessions**	23.78 ± 12.75 ms	53.61%		
TOJc	Short(1)	30.65 ± 14.33 ms	46.77%	35.93%	0.74
	Short(2)	44.63 ± 19.59 ms	43.88%		
	Long	29.51 ± 22.07 ms	74.81%		
	**All sessions**	34.93 ± 19.74 ms	56.52%		

*As noted in the Methods, the initial comparison stimulus in the nAD task was inadvertently set incorrectly. The ICC marked with an asterisk is the ICC when including Short(1) and Short(2) measurements only.

**Table 2:** Descriptive statistics of results from the vibrotactile tasks using the truncated Long version.

Task	Session	Mean ± SD	CV_bs_	CV_ws_	ICC
sRT	*See* Table 1 *for Short(1) and Short(2) results*			11.79%	0.91
	Long (trunc.)	224.73 ± 68.04 ms	30.27%		
	**All sessions**	218.47 ± 55.96 ms	25.61%		
sRT (ISV)	*See* Table 1 *for Short(1) and Short(2) results*			26.40%	0.77
	Long	45.28 ± 20.97 ms	46.30%		
	**All sessions**	47.79 ± 19.21 ms	40.21%		
cRT	*See* Table 1 *for Short(1) and Short(2) results*			9.99%	0.88
	Long (trunc.)	406.23 ± 77.57 ms	19.10%		
	**All sessions**	423.98 ± 94.08 ms	22.19%		
cRT (ISV)	*See* Table 1 *for Short(1) and Short(2) results*			26.77%	0.56
	Long	76.31 ± 30.85 ms	40.42%		
	**All sessions**	74.09 ± 28.22 ms	38.09%		
sDT	*See* Table 1 *for Short(1) and Short(2) results*			43.36%	0.34
	Long (trunc.)	2.45 ± 0.97 μm	39.64%		
	**All sessions**	4.15 ± 1.89 μm	45.43%		
dDT	*See* Table 1 *for Short(1) and Short(2) results*			14.93%	0.67
	Long (trunc.)	7.66 ± 1.31 μm	17.06%		
	**All sessions**	8.22 ± 1.87 μm	22.80%		
nAD	*See* Table 1 *for Short(1) and Short(2) results*			38.63%	0.56
	Long (trunc.)	47.71 ± 14.67 μm	30.75%		
	**All sessions**	41.09 ± 18.59 μm	45.24%		
dAD	*See* Table 1 *for Short(1) and Short(2) results*			68.28%	0.30
	Long (trunc.)	10.80 ± 15.43 μm	142.90%		
	**All sessions**	17.81 ± 13.92 μm	78.12%		
sAD	*See* Table 1 *for Short(1) and Short(2) results*			54.57%	<0.07
	Long (trunc.)	19.87 ± 15.48 μm	77.93%		
	**All sessions**	40.80 ± 27.10 μm	66.42%		
sqFD	*See* Table 1 *for Short(1) and Short(2) results*			57.14%	<0.01
	Long (trunc.)	3.61 ± 3.38 Hz	93.41%		
	**All sessions**	5.90 ± 3.25 Hz	55.04%		
smFD	*See* Table 1 *for Short(1) and Short(2) results*			51.30%	0.30
	Long (trunc.)	4.48 ± 3.30 Hz	73.64%		
	**All sessions**	6.94 ± 3.73 Hz	53.69%		
TOJs	*See* Table 1 *for Short(1) and Short(2) results*			33.30%	0.72
	Long (trunc.)	37.44 ± 17.02 ms	45.46%		
	**All sessions**	33.67 ± 17.94 ms	53.29%		
TOJc	*See* Table 1 *for Short(1) and Short(2) results*			34.09%	0.62
	Long (trunc.)	40.33 ± 22.32 ms	55.35%		
	**All sessions**	38.40 ± 19.24 ms	50.11%		

### Reaction time

**Figure 4:**
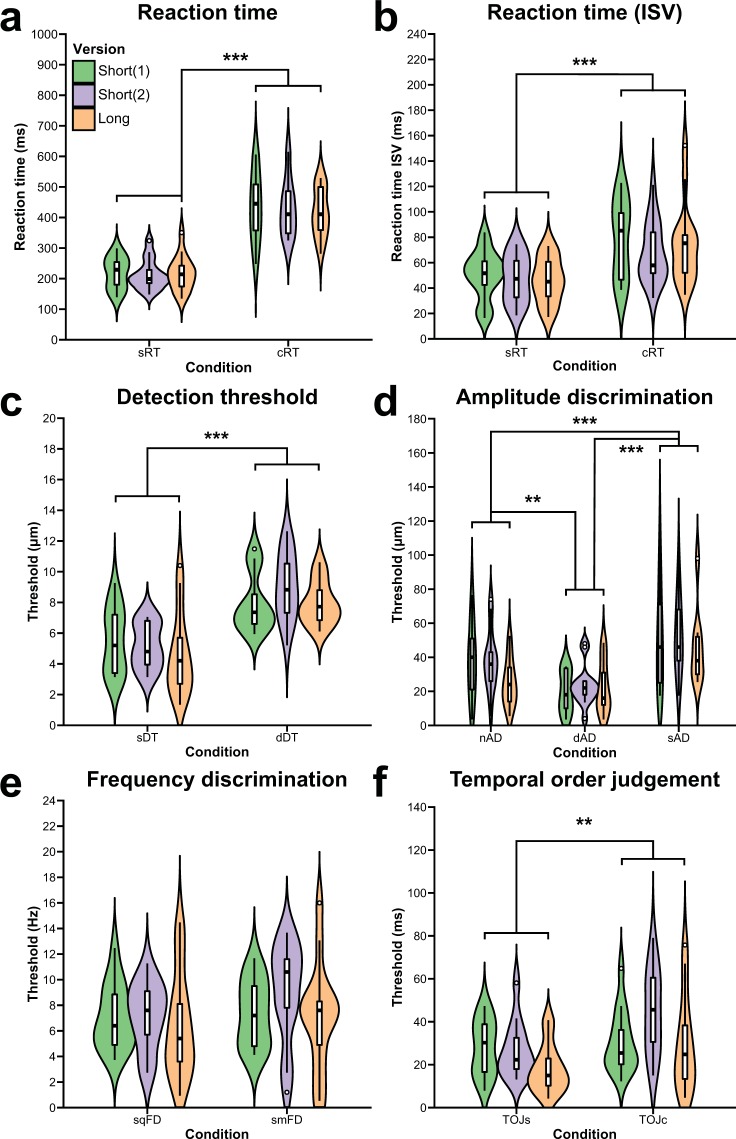
Violin plots overlaid by boxplots displaying the distribution of vibrotactile detection and discrimination thresholds for each paradigm and for each version of each paradigm. Results for (**a**) reaction time; (**b**) intrasubject variability (ISV) of reaction time; (**c**) detection threshold; (**d**) amplitude discrimination; (**e**) frequency discrimination; and (**f**) temporal order judgment are shown. sRT, simple reaction time; cRT, choice reaction time; sDT, static detection threshold; dDT, dynamic detection threshold; nAD, amplitude discrimination with no adaptation; dAD, amplitude discrimination with dual-site adaptation; sAD, amplitude discrimination threshold with single-site adaptation; sqFD, sequential frequency discrimination; smFD, simultaneous frequency discrimination; TOJs, temporal order judgment without carrier stimulus; TOJc, temporal order judgment with carrier stimulus. **p < 0.01; ***p < 0.001; Holm-Bonferroni correction was applied on the amplitude discrimination threshold comparisons.

The mean reaction times across the three sessions for the sRT and cRT tasks were 215.45 ± 51.13 ms and 427.60 ± 93.81 ms, respectively. The CV_bs_ across all sessions were 23.7% and 21.9% and the CV_ws_ were 10.9% and 9.5%, respectively. The ICCs were 0.90 and 0.90. Truncating the long versions of these tasks resulted in ICCs of 0.91 and 0.88. No task showed a significant time or version effect. Reaction times for cRT were shown to be significantly longer compared to the reaction times for sRT [χ^2^(1) = 136.22, *p* < 0.001; effect size = 0.84], as shown in Fig. 4a. The mean ISV across the three sessions for the sRT and cRT tasks were 48.05 ± 18.07 ms and 73.17 ± 28.24 ms. The CV_bs_ across all sessions were 37.6% and 38.6% and the CV_ws_ were 23.4% and 26.5%, respectively. The ICCs were 0.78 and 0.63. Truncating the long versions of these tasks resulted in ICCs of 0.77 and 0.56. There were no significant version or time effects for either task. The ISV for cRT was significantly greater compared to the ISV for sRT [χ^2^(1) = 29.97, *p* < 0.001; effect size = 0.33] (Fig. 4b).

### Detection threshold

The mean detection thresholds across the three sessions for the sDT and dDT tasks were 5.10 ± 2.14 μm and 8.32 ± 1.87 μm, respectively. The CV_bs_ across all sessions were 42.0% and 22.5% and the CV_ws_ were 30.9% and 13.5%, respectively. The ICCs were 0.65 and 0.78. Truncating the long versions of these tasks resulted in ICCs of 0.34 and 0.67. There were no significant version or time effects for either task. As shown in Fig. 4c, dDTs were significantly higher than sDTs [χ^2^(1) = 54.64, *p* < 0.001; effect size = 0.57].

### Amplitude discrimination

The mean AD threshold across the three sessions for the nAD, dAD and sAD tasks were 34.14 ± 18.81 μm, 21.95 ± 12.69 μm and 49.73 ± 24.68 μm, respectively. The CV_bs_ across all sessions were 55.1%, 57.8% and 49.6% and the CV_ws_ were 40.5%, 45.7% and 31.4%, respectively. The ICCs were 0.44, 0.53 and 0.72. Truncating the long versions of these tasks resulted in ICCs of 0.56, 0.30 and 0.07. Only the nAD task showed a significant version effect [χ^2^(1) = 5.53, *p* = 0.02; effect size = 0.17]; no task showed a significant time effect. After accounting for the version effect in the nAD task, a significant difference in AD thresholds was found [χ^2^(2) = 42.84, *p* < 0.001; effect size = 0.30]. Post hoc pairwise comparisons showed that dAD thresholds were significantly lower than nAD (*z* = −3.06, *p*_Holm_ = 0.002) and sAD thresholds (*z* = 7.14, *p*_Holm_ < 0.001) (Fig. 4d). In addition, nAD thresholds were significantly lower than sAD thresholds (*z* = 4.07, *p*_Holm_ < 0.001).

### Frequency discrimination

The mean FD thresholds across the three sessions for the sqFD and smFD tasks were 6.85 ± 3.08 Hz and 7.82 ± 3.61 Hz, respectively. The CV_bs_ across all sessions were 45.0% and 46.2% and the CV_ws_ were 41.7% and 37.0%, respectively. The ICCs were 0.25 and 0.43. Truncating the long versions of these tasks resulted in ICCs of < 0.01 and 0.30. There were no significant version or time effects for either task. No significant difference was found between the two tasks (effect size = 0.03) (Fig. 4e).

### Temporal order judgment

The mean TOJ thresholds across the three sessions for the TOJs and TOJc tasks were 23.78 ± 12.75 ms and 34.93 ± 19.74 ms, respectively. The CV_bs_ across all sessions were 53.6% and 56.5% and the CV_ws_ were 46.0% and 36.0%, respectively. The ICCs were 0.07 and 0.74. Truncating the long versions of these tasks resulted in ICCs of 0.72 and 0.62. Only the TOJs task showed a significant version effect [χ^2^(1) = 5.27, *p* = 0.02; effect size = 0.17]; no task showed a significant time effect. After accounting for the version effect in the TOJs task, TOJc thresholds were found to be significantly higher than TOJs thresholds [χ^2^(1) = 10.45, *p* = 0.001; effect size = 0.13], as shown in Fig. 4f.

### Convergence

The mean convergence score was 3.42 ± 1.05 across all tasks. While there was a significant task effect [χ^2^(7) = 31.36, *p* < 0.001; effect size = 0.09], there was no significant version effect [χ^2^(1) = 0.05, *p* = 0.83; effect size = 0.00] or a time effect [χ^2^(1) = 0.98, *p* = 0.32; effect size = 0.00]. There was also no significant task × version effect [χ^2^(8) = 4.10, *p* = 0.85; effect size = 0.01] or task × time effect [χ^2^(8) = 3.66, *p* = 0.89; effect size = 0.00]. Post hoc pairwise comparisons showed that convergence scores were significantly lower for the simultaneous frequency discrimination (*z* = −4.87, *p*_Holm_ < 0.001) and temporal order judgement (*z* = −4.13, *p*_Holm_ = 0.001) paradigms compared to the static detection protocol. Convergence scores were also significantly lower for the simultaneous frequency discrimination paradigm compared to the amplitude discrimination with single-site adaptation (*z* = −3.28, *p*_Holm_ = 0.03) and dual-site adaptation paradigms (*z* = −3.28, *p*_Holm_ = 0.03). The convergence scores were otherwise comparable between paradigms.

## Discussion

In this study, the test-retest reliability of vibrotactile detection and discrimination thresholds was assessed for the first time. As has been shown extensively in other behavioural studies^50–52^, simple and choice reaction times were highly reproducible. Additionally, the ISV of subjects’ reaction times was shown to have excellent test-retest reliability, a finding observed in other domains and modalities^53,54^. The reproducibility of the remaining measures (detection thresholds, amplitude discrimination, frequency discrimination, and TOJ) is less well-documented; this study presents some of the first evidence that these measures are reproducible to a variable extent. Moreover, the intersubject variability of the various thresholds falls in line with previous findings in adults^13,27–29^, with reaction times having around 20–30% variation, detection and frequency discrimination thresholds having 20–40% variation, and amplitude discrimination thresholds having 40–60% variation. The intrasubject variability of detection thresholds has been shown to be around ~25–30%^55,56^, which also falls in line with the present findings.

Notwithstanding the findings of this study, the interpretation of ICCs must be considered carefully, given that an ICC is a ratio of between-subject variability and the sum of between-subject variability and error—pointing to its sensitivity to interindividual differences. In clinical settings, it would be worthwhile to consider individuals’ inherent ability to perform each task. For instance, some participants may exhibit increased trial-to-trial variability that reflects specific pathological processes, rather than pointing toward an unreliable psychophysical measure of vibrotactile processing per se. An analysis of individual differences in detection and discrimination performance was beyond the scope of this study but is a potential avenue for future research. There is also the matter of what is considered a “good” ICC for clinically relevant behavioural measures. Based on Cicchetti’s guidelines^57^, we conclude that the vibrotactile reaction times have excellent reliability, the ISV of reaction times has good-to-excellent reliability, detection thresholds have good-to-excellent reliability, amplitude discrimination thresholds have fair-to-good reliability, frequency discrimination thresholds have poor-to-fair reliability, and TOJ has good reliability. The poorer reliability of the frequency discrimination thresholds can be explained in part by the difficulty of the task. As reported previously^13^, many participants stated that they were not able to discriminate between the two frequencies, with some having to repeat the practice trials multiple times to proceed to the testing component.

Task-related effects that have been shown previously were also replicated in this study. Differences between simple and choice reaction times are a well-established effect^58^, so it is unsurprising to see this replicated in this study. Significantly higher dynamic detection thresholds compared to static detection thresholds^13,17,19,20,23–25^, higher single-site-adaptation amplitude discrimination thresholds^13,17,19,23–25,59^ and lower dual-site-adaptation amplitude discrimination thresholds^59^ compared to no-adaptation amplitude discrimination thresholds, and higher TOJ thresholds with a carrier stimulus compared to simple TOJ thresholds^17,22,24,60^ also appear to be consistent effects in healthy, clinical, younger and older cohorts, demonstrating high reproducibility across the general population. However, this study failed to reproduce the effect of higher frequency discrimination thresholds when stimuli are simultaneously delivered rather than being sequentially delivered despite previous evidence of this^13,23,24^. The within- and between-subject variability of each of these measures will directly influence the likelihood of observing a differential effect in experimental designs (e.g., comparing measures from a clinical cohort and healthy controls). It is worth noting that increased measurement and participant variability could point toward specific somatosensory deficits, and more importantly, heterogeneity of phenotypes in clinical disorders such as autism spectrum^61,62^ and attention-deficit hyperactivity disorders^63,64^. To reiterate, systematic probing of individual differences in vibrotactile detection and discrimination thresholds would provide a different perspective on somatosensory (dys)function.

The tasks applied in this battery have been strongly linked to neurophysiological mechanisms, particularly those related to inhibitory function. Static detection threshold and its dynamic counterpart, in particular, have been linked to feed-forward inhibition and sensory gating. The significantly higher dynamic detection thresholds replicated in this study follow the theory that the ramping up of a stimulus from an undetectable level leads to increased thresholds as a result of initial adaptation from inhibitory interneurons, which have a lower spiking threshold^25,33^. The differential effects of single- and dual-site adaptation on amplitude discrimination—also replicated in this study—reflect the engagement of lateral inhibition that either enhances or diminishes the ability to distinguish between stimuli^59,65^. This is supported by the lack of such effects in ASD^17,66^, where inhibition is thought to be dysfunctional. Additionally, prior work has linked aberrant vibrotactile processing to abnormal GABA levels in the brain^19,23,29^. High ICCs suggest that vibrotactile thresholds as a measure are very stable in a healthy population and thus are useful in determining abnormal cortical physiology as well.

Psychophysical experiments historically involve hundreds of trials and low subject numbers. The vibrotactile battery used here was originally designed so that it could be completed within 50–60 min, making it suitable for a paediatric cohort. While this is somewhat atypical in psychophysical terms, there were no significant effects of battery length for most of the tasks, signifying that the original length of the battery is appropriate (at the very least for adults). Moreover, the relatively short length makes it more practical to collect data from a higher number of participants, which would counter the lower reliability of some of the vibrotactile measures.

While there did not appear to be any effects of training or battery length, several factors that may influence reproducibility were not taken into account. For instance, attentional effects were not systematically controlled for or assessed. There is evidence that attention can enhance the cortical dynamics of tactile processing^67,68^. Another possible drawback of this study was that was the order of tasks was kept fixed in each session. The less reproducible and ostensibly more difficult tasks (frequency discrimination) were always toward the end of the session, meaning fatigue effects could have played a detrimental effect on test-retest reliability. To counter such possible systematic effects, future implementations of vibrotactile paradigms would benefit from randomizing the presentation order of tasks. Additional effects not controlled for in the current study were caffeine consumption, sleep, and menstrual cycle, which could lead to different results in different sessions as well. We did, however, test whether participants could use audible cues to aid their performance (as was reported for a different device^69^), but this was not the case.

In conclusion, vibrotactile detection and discrimination thresholds show good reproducibility. This study lends further support to the value of psychophysical approaches to probe tactile function in an array of human populations.

## Data Availability

Raw data, R markdown files, and supplementary figures generated in this study are publicly available on the Open Science Framework: 10.17605/OSF.IO/5ZA9F.
